# Supplementary material for: Mental Health Status of New Police Trainees before and during the COVID-19 Pandemic
Source: Healthcare (Basel). 2024 Mar 13;12(6):645. doi: 10.3390/healthcare12060645 (PMC11154500; doi:10.3390/healthcare12060645)
Supplement: Supplementary file 1 [file healthcare-12-00645-s001.zip › healthcare-2865563-supplementary.pdf]

Supplementary Table S1. Difference prevalence after PSM

N=4,824 (2019, 2020)		Insomnia			Depression			Anxiety		
		**2019**	**2020**	**Difference**	**2019**	**2020**	**Difference**	**2019**	**2020**	**Difference**
		**(N=2,412)**	**(N=2,412)**	**(95%CI)**	**(N=2,412)**	**(N=2,412)**	**(95%CI)**	**(N=2,412)**	**(N=2,412)**	**(95%CI)**
Total		2	2.9	0.9*	0.4	1.3	0.9*	1.2	3.2	2.0*
		(1.5-2.7)	(2.3-3.7)	(0.0-1.8)	(0.2-0.7)	(0.8-1.8)	(0.4-1.4)	(0.8-1.7)	(2.5-4.0)	(1.2-2.8)
Sex	men	1.7	2	2.9	0.2	0.8	0.6*	0.6	1.7	1.0*
		(1.2-2.4)	(1.4-2.8)	(-0.6-1.2)	(0.0-0.5)	(0.4-1.3)	(0.2-1.1)	(0.3-1.1)	(1.1-2.4)	(0.3-1.7)
	women	2.9	5.6	2.7*	0.9	2.4	1.5*	2.6	7.1	4.6*
		(1.8-4.5)	(4.0-7.7)	(0.5-4.9)	(0.3-1.9)	(1.4-3.9)	(0.1-2.9)	(1.5-4.1)	(5.3-9.4)	(2.2-6.9)
Age group	20-29	2.2	2.9	0.7	0.4	1.3	0.8*	1.1	3.1	2.0*
		(0.6-2.9)	(2.3-3.8)	(-0.2-1.7)	(0.2-0.9)	(0.8-1.8)	(0.3-1.4)	(0.7-1.8)	(2.4-4.0)	(1.1-2.9)
	≥30	1.5	3.3	1.8	0.2	1.1	0.9	1.2	3.3	2.1
		(0.7-2.9)	(1.5-6.1)	(-0.6-0.4)	(0.0-0.9)	(0.2-3.2)	(-0.4-2.2)	(0.5-2.4)	(1.5-6.1)	(-0.2-0.4)
Education group	< College degree	2.3	2.5	0.2	0.2	0.6	0.3	0.9	2.3	1.4*
		(1.5-3.3)	(1.7-3.4)	(-1.0-1.4)	(0.0-0.7)	(0.2-1.1)	(-0.1-0.8)	(0.4-1.5)	(1.3-3.3)	(0.5-2.4)
	≥ College degree	1.7	3.7	1.9*	0.5	2.2	1.7*	1.5	4.3	2.8*
		(1.1-2.7)	(2.6-5.0)	(0.5-3.3)	(0.2-1.1)	(1.4-3.3)	(0.7-2.7)	(0.9-2.4)	(3.1-5.7)	(1.4-4.2)
Monthly household income	< 4,000 USD	2.3	3.8	1.5*	0.2	1.7	1.5*	1.1	4.7	3.6*
		(1.6-3.3)	(2.8-5.1)	(0.1-2.9)	(0.1-0.7)	(1.0-2.6)	(0.7-2.3)	(0.6-1.9)	(3.6-6.1)	(2.1-4.8)
	≥ 4,000 USD	1.8	2.2	0.4	0.5	0.2	0.3	1.2	1.7	0.5
		(1.1-2.7)	(1.4-3.2)	(-0.7-1.5)	(0.2-1.1)	(0.4-1.5)	(-0.4-0.9)	(0.7-2.1)	(1.1-2.6)	(-0.4-1.4)

* PSM with sex age group, education levels and monthly household income
